# Identifying large sets of unrelated individuals and unrelated markers

**DOI:** 10.1186/1751-0473-9-6

**Published:** 2014-03-17

**Authors:** Kuruvilla Joseph Abraham, Clara Diaz

**Affiliations:** 1 Departamento do Biologia Celular e Molecular, Faculdade da Medicina, Universidade de São Paulo, Ribeirão Preto, Brazil; 2 Dpto. Mejora Genètica Animal. INIA. Ctra. de la Coruña km.7.5 28040, Madrid, Spain; 3 Departamento de Puericultura e Pediatria, Faculdade da Medicina, Universidade de São Paulo, Ribeirão Preto, Brazil

## Abstract

**Background:**

Genetic Analyses in large sample populations are important for a better understanding of the variation between populations, for designing conservation programs, for detecting rare mutations which may be risk factors for a variety of diseases, among other reasons. However these analyses frequently assume that the participating individuals or animals are mutually unrelated which may not be the case in large samples, leading to erroneous conclusions. In order to retain as much data as possible while minimizing the risk of false positives it is useful to identify a large subset of relatively unrelated individuals in the population. This can be done using a heuristic for finding a large set of independent of nodes in an undirected graph. We describe a fast randomized heuristic for this purpose. The same methodology can also be used for identifying a suitable set of markers for analyzing population stratification, and other instances where a rapid heuristic for maximal independent sets in large graphs is needed.

**Results:**

We present FastIndep, a fast random heuristic algorithm for finding a maximal independent set of nodes in an arbitrary undirected graph along with an efficient implementation in C++. On a 64 bit Linux or MacOS platform the execution time is a few minutes, even with a graph of several thousand nodes. The algorithm can discover multiple solutions of the same cardinality. FastIndep can be used to discover unlinked markers, and unrelated individuals in populations.

**Conclusions:**

The methods presented here provide a quick and efficient method for identifying sets of unrelated individuals in large populations and unlinked markers in marker panels. The C++ source code and instructions along with utilities for generating the input files in the appropriate format are available at http://taurus.ansci.iastate.edu/wiki/people/jabr/Joseph_Abraham.html

## Background

The analysis of genotypes collected from large numbers of individuals is necessary for Genome Wide Asociation Studies (GWAS), conservation biology, and population genetics among other purposes. Most statistical analyses in these areas assume the individuals are unrelated, which may not always be the case in large sample populations. One way to avoid false positives due to the presence of related individuals is to identify the largest subset of unrelated individuals in the study population and retain only those individuals. A similar computational issue arises when identifying sets of unrelated markers for use in analyzing population stratification. Analyzing population stratification with the dense marker maps which are now currently available can be very time consuming due to the large number of markers being used, however due to linkage disequilibrium between the markers in dense maps not all the information provided by the markers can be considered independent. In this situation it makes sense to select a subset of markers which are mutually independent and which can provide sufficient information to analyze population stratification; this strategy reduces the computational burden arising from using all available markers while attempting to retain as many markers as possible. Indeed, certain well established approaches to analyzing population stratification assume that the input markers are unrelated [1], thus there are both conceptual and computational reasons for using a subset of weakly correlated markers. However, in populations with a complex history, identifying this set of markers may not be possible using just genetic map information, but may also require a more detailed analysis which takes into account the patterns of linkage disequilibrium between markers. In this manuscript we show how these two problems are related and present methods which address both problems.

In order to proceed with identifying a large subset of unrelated individuals it is important to quantify relatedness between individuals; one way to do this is to use genotype information to assess Identity by State (IBS) which in turn can be used to estimate pairwise Identity by Descent (IBD), for example by using a Hidden Markov Model as is done in PLINK [2]. This is not the only possibility, in [3] genotype information is used to find an estimator for the coefficient of kinship between individuals which is used as a measure of relatedness. Other possibilities for relating allele sharing to similarities between individuals are discussed in [4] and [5]. Once the pairwise IBD (or some other similarity measure) is known for all pairs of individuals in the population, we can assemble a symmetric matrix of distances between individuals. As has been pointed out in [6], once a suitable threshold for defining unrelatedness has been specified it becomes possible to define an undirected graph whose nodes correspond to individuals with edges connecting unrelated individuals. This is not the only possibility, it is also possible to define a graph where edges are present between individuals who are related. Both definitions lead to graphs whose structure can be analyzed to extract subsets of mutually unrelated individuals. FastIndep makes use of a graph in which related individuals are connected by edges. Regardless of how the graph is specified a threshold must be defined, and the value of this threshold will depend on the similarity measure used. For example in [3], it is shown how the estimate for the coefficient of kinship along with the information on the number of markers can be used to fix this threshold and thus the edge structure of the graph. The graph, in addition to being undirected is also unweighted, *i.e.* the actual extent of similarity between the individuals is not as important as whether or not the similarity falls above or below a certain user defined threshold. For addressing the problem of finding a large number of unlinked markers, the undirected graph arises from assigning a node to each marker and an edge between two nodes if the linkage disequilibrium between the corresponding markers is above some threshold for statistical significance as defined by the user.

Finding a large number of mutually unrelated individuals or mutually unrelated markers corresponds to finding a large subset of the nodes in the graph such that no two nodes in the subset are connected by one of the edges in the graph. Such subsets of nodes constitute independent sets, and for our purposes we seek maximal independent sets *i.e.* independent sets with the property that there exists no other node in the graph which can be added to the independent set while retaining the property that all nodes remain mutually unrelated. In graphs with sizes corresponding to real data there may be many maximal independent sets and the question which arises is, which one of these to choose. Frequently, we wish to maximize the number of markers or unrelated individuals. This requires finding the largest maximal independent set present in the graph. The maximal independent set of largest size is the maximum independent set, and in many graphs there may be more than one maximum independent set. Finding the maximum independent set is NP-hard, *i.e.* there is no known efficient algorithm for finding the maximum independent set for an arbitrary graph. That is why exact algorithms, such as the Bron Kerbosch algorithm [7] which can be used to find all maximal independent sets (including maximum independent sets) on an arbitrary graph, are prohibitively slow for graphs beyond a certain size. This necessitates the use of heuristics for problems with realistic sizes, for reasons which will be clear later FastIndep uses a stochastic heuristic. The need for heuristics has been recognized in earlier work [6] where the use of the Bron Kerbosch algorithm is restricted to small graphs with upto a few hundred nodes at most, and for larger graphs a deterministic heuristic algorithm which outputs a single maximal independent set is used.

The FastIndep algorithm was first introduced for selecting unlinked markers for analyzing population stratification and was first discussed in [8]. FastIndep differs from the Primus software of [6] in a number of respects most notably in that the FastIndep algorithm is a stochastic greedy heuristic regardless of the size of the problem. The use of a stochastic heuristic is motivated by [9] where it is demonstrated that randomization may improve on deterministic heuristics for graph triangulations arising in genetic linkage analysis. Furthermore as pointed out in [6], when analyzing a population containing both healthy individuals and those affected with some disease it may be useful to consider maximal independent sets containing a large number of affected individuals; such sets may or may not be maximum independent sets. If the graph is sufficiently large, then it may not be possible to use the Bron Kerbosch algorithm to enumerate all maximal independent sets and pick the most suitable one. In this situation, it may be useful to work with a stochastic heuristic which outputs a number of different maximal independent sets, one or more of which can be choosen. A deterministic heuristic by contrast may not offer the possibility of easily checking for alternatives.

Another advantage to using a stochastic heuristic arises when selecting a subset of markers for analyzing population stratification. If the original set of markers is sufficiently large it is not feasible to use the exact Bron Kerbosch algorithm to select the largest subset of unrelated markers, necessitating the use of some approximate algorithm. This raises the question of the dependence of the final results on the choice of markers. One way to check the extent to which the final results are dependent on the choice of markers is to repeat the analysis of population stratification using alternative sets of markers generated by running the algorithm repeatedly. If the results from using different sets of markers are consistent then the variation of the final results with the choice of markers should be quite small. This consistency check is unique to FastIndep and is not available in other publicly available codes for finding maximal independent sets. The FastIndep code is sufficiently general that it is not restricted to a particular choice of correlation measure, all that matters is that the larger the entry in the matrix, the stronger the correlation between the corresponding individuals (or markers). Any correlation measure that satisfies this criterion may be used so long as the entries of the matrix are larger than or equal to zero. With different correlation measures different thresholds for independence may be required; it is the responsibility of the user to define these thresholds depending on the correlation measure used. For example analysing a marker panel using the Linkage Disequilibrium measure *r*^2^ the threshold could be choosen based on the relation between the *r*^2^ and the Pearson Correlation coefficient and thus the *χ*^2^ distribution. For finding unrelated individuals using the method of [3] the threshold would be fixed based on the sampling distributions for the coancestry described in [3].

## Methods

The current C++ implementation of FastIndep is closely related to the one described in [8] for the purpose of selecting a large subset of unlinked markers for analyzing population stratification and will be briefly described below. The key idea behind the algorithm is to start with a deterministic greedy heuristic (which typically finds a reasonably large maximal independent set), and then randomize the heuristic in order to explore solutions close to the one found by the deterministic algorithm. The algorithm is run many times, and due to the stochastic nature of the algorithm the output from the different runs will contain a variety of different maximal independent sets. Some of these maximal independent sets may be larger than the one uncovered by the deterministic algorithm and may not be easy to discover without the randomization, which is the motivation behind introducing a stochastic element to the algorithm. Furthermore, if the algorithm is efficiently implemented, the additional time needed for the multiple runs is not excessive. We emphasize that the algorithm does not make use of map information, this lack of dependence on map information allows the application of the algorithm to genetic data with very complex patterns of Linkage Disequilbrium, as well as to problems where there is no analogue of map information, such as identifying large numbers of unrelated individuals in populations. A description of the algorithm follows, although the same algorithm can be used for identifying both unlinked markers and unrelated invididuals, the description will be in terms of finding unlinked markers. The rest of the section can safely be skipped without any loss of continuity by readers not interested in technical details of the FastIndep algorithm.

As before we assume we have an undirected graph in which is the set of nodes, ={*V*_*i*_} for *i* ≤ *i* ≤ *n*, and is the set of edges. is not assumed related to any genetic marker map so the algorithm is at this stage perfectly general. In the most general case, the algorithm also requires as input a positive parameter *γ* which is a measure of how far the algorithm deviates from a deterministic greedy heuristic. The larger the value of *γ* the closer the algorithm resembles a deterministic heuristic. In the implementation presented here *γ* is set equal to one. We define *D*_*i*_ the set of non neighbors of *V*_*i*_ and *n*_*i*_ size of *D*_*i*_, and consider only nodes for which *n*_*i*_ > 0, *i.e.* we ignore in the discussion nodes connected to all other nodes. This corresponds to ignoring markers in tight Linkage Disequilibrium with all other markers in the panel. We define sets of nodes *CandSet*, *TempSet* as well as *ReturnSet*, which is the output from the program. *ReturnSet* is initialized by the set of all nodes not connected to any other nodes. The algorithm is initialized by selecting a node disconnected to many others, with a probability which depends on how few neighbors it has. 

• Initialization 

1. Evaluate normalized $p_{i}=(n_{i}^{\gamma }/\text{norm})\;\;\forall \;i\;$ where $\text{norm}=\underset{i=1}{\sum }\;n_{i}^{\gamma }$

2. Pick some *V*_*j*_ with probability *p*_*j*_ and insert in *ReturnSet*.

3. *CandSet* ← *D*_*j*_.

4. *TempSet* ← *∅*.

• Main Loop

 while *CandSet* ≠ *∅* do 

1. Select some *V*_*k*_ ∈ *CandSet* with probability *p*_*k*_ normalized over *CandSet* to insert in *ReturnSet*

2. *TempSet* ← {*V*_*m*_ ∈ *D*_*k*_ : *V*_*m*_ ∉ *ReturnSet* where 1 ≤ *m* ≤ *N*}

3. *CandSet*←(*CandSet* ∩ *TempSet*)

 If *CandSet* ≡ *∅* return *ReturnSet*.

In step 1 of the initialization, the normalized *p*_*i*_ are computed for all nodes except nodes which are connected to all others or disconnected to all others. Nodes disconected to all others are automatically included in the final output, while nodes connected to all others do not contribute to maximal independent sets of any interest for our purposes. This leads to a substantial speed up of the program with little loss of information.

It is also possible to define a deterministic greedy heuristic in which the nodes selected in step 2 of the initialization and step 1 of the main loop are just those with the largest associated probability.

## Results and discussions

The input to FastIndep consists of a carefully formatted symmetric matrix of positive correlation values (which may exceed one in value), a threshold value taking values between 0 and 1, and an integer specifying the number of times the algorithm is to be run. The input has a structure similar to a dataframe in the R programing language [10], where the column names are the names of the individuals or markers and the row names are the same as the column names. An undirected graph is constructed by assigning a node to each individual and then declaring that edges exist between nodes provided that the correlation between the individuals is greater than the user specified threshold value. As the algorithm is stochastic in nature running the algorithm repeatedly may occasionally yield the same maximal independent set more than once, thus the output of the algorithm is written to a text file which lists only the unique independent sets found. Included in the output of FastIndep is the result from the deterministic greedy heuristic discussed earlier. In all results presented, the random numbers used by FastIndep are generated using the methods of [11].

### Results on simulated data

In order to to check the size distribution of the maximal independent sets found by FastIndep, we generated a total of 6 graphs, 2 of size 500, 2 of size 1000, and 2 of size 2000, by using a U(0,1) random number generator to generate symmetric random matrices which were then transformed into graphs using two given thresholds to define edges. The size distribution of the maximal independent sets found by FastIndep on these graphs is shown in Table 1.

**Table 1:** Quantile distribution of set sizes for artificial data set

**Graph size**	**Threshold**	**Minimum**	**25%**	**Median**	**75%**	**Maximum**
500	0.15	3	3	4	4	6
500	0.95	53	63	65	67	77
1000	0.15	3	4	4	5	7
1000	0.95	66	75	78	80	91
2000	0.15	3	4	5	5	7
2000	0.95	78	89	91	93	104

Edges generated by thresholding values generated at random (U(0,1)).

In order to test the capacity of FastIndep to find multiple maximum independent sets in graphs of smaller sizes we generated a total of 3111 graphs of sizes ranging from 10 to 60 using a U(0,1) random number generator to generate symmetric random matrices which were then transformed into graphs using a threshold to define edges, 61 thresholds were used for each graph size. The number of runs in FastIndep was choosen to be 1000 times the number of nodes for each graph. In all cases the largest maximal independent sets found by FastIndep are the same size as those found using a branch and bound algorithm, [12,13], and in 2597 cases FastIndep found multiple largest cardinality sets. This indicates the capacity of FastIndep to discover multiple maximal independent sets, a feature which is valuable when considering real data.

We next use simulated data to compare FastIndep with three other algorithms used in statistical genetics Primus, KING [14] and PLINK [2]. Both KING and PLINK are implemented in the Primus software, and it is these implmentations we will use. To make the comparison with KING we note that the algorithm in KING is simply the deterministic greedy heuristic which is the starting point for FastIndep. Since the output of FastIndep includes the result from the deterministic greedy heuristic as well as all results from the stochastic heuristic, some of which which may be larger than the result of the greedy heuristic, FastIndep either performs as well as KING or outperforms KING on any input dataset.

In order to compare FastIndep with Primus and PLINK a total 50 graphs were generated with sizes ranging from 900 to 1300 and thresholds ranging from 0.05 to 0.95 using the same procedure as before. These sizes were choosen to be comparable with those of modern genetic analyses which may involve thouands of individuals. The number of runs of FastIndep was choosen to be 50 times the number of nodes in the graph. The total running time for all fifty graphs for FastIndep was 3142 seconds running on a Linux workstation with 36 GB of ram on a 3.2 GHz processor running GCC 4.7.2. On the same platform with perl 5 version 14, Primus required 20561 seconds. Nonetheless in only three cases the maximal independent set found by FastIndep was smaller than that found by Primus. In 35 of other trials the maximal independent set found by FastIndep was larger that that found by Primus. In 12 of the random graphs, FastIndep found multiple maximal independent sets with the same cardinality as those found by Primus. The results of this comparison are summarized in Table 2, where the maximal independent set sizes found by FastIndep and Primus are shown, with the Primus results in brackets. Based on the results of Table 2 FastIndep appears to perform as well as or better than Primus except for a few graphs with a large threshold; such graphs tend to be sparse. The difference in sizes between the maximal independent sets found by FastIndep and Primus are sumarized in Table 3. Positive values indicate larger maximal independent set sizes found by FastIndep. The same set of graphs were used to compare FastIndep and PLINK, based on the results of [6] where it was shown that Primus outperforms PLINK, it would be expected that FastIndep would considerably outperform PLINK. This was indeed the case.

**Table 2:** Comparing FastIndep with Primus

	**0.05**	**0.15**	**0.25**	**0.35**	**0.45**	**0.55**	**0.65**	**0.75**	**0.85**	**0.95**
900	4(4)	7(7)	8(8)	10(9)	12(11)	15(14)	19(17)	25(24)	39(36)	94(96)
1000	4(4)	6(6)	8(8)	10(9)	12(12)	15(14)	19(17)	26(23)	39(34)	93(87)
1100	5(4)	7(6)	8(8)	10(9)	13(11)	15(14)	20(16)	27(25)	39(37)	93(94)
1200	5(4)	6(6)	8(8)	10(9)	12(11)	15 (14)	20(16)	26(23)	40(39)	95(98)
1300	5(4)	7(6)	8(8)	10(10)	12(11)	16(14)	21(18)	26(24)	41(38)	97(96)

Set sizes from FastIndep and (Primus). Graph Sizes along Rows & Thresholds along columns.

**Table 3:** Distribution of differences between Primus and FastIndep

**Minimum**	**25%**	**Median**	**75%**	**Maximum**
-3	0	1	2	6

Difference in Maximal Independent Set Sizes found by FastIndep and Primus.

Recently, a new algorithm LEAF [15] was introduced to find maximal independent sets on graphs which arise from protein sequence comparison; as pointed out in [15] such graphs are typically sparse. In the context of finding unrelated individuals in a study population whose members are choosen to be distantly related or unrelated, the sparsity assumption may be valid; however in the context of finding subsets of unrelated markers a sparse graph would correspond to relatively little Linkage Disequilibrium between markers, which is a needlessly restrictive assumption. More particularly, the capacity of of a handfull of haplotype tagging SNPs in genetic marker maps to tag a large fraction of the markers present [16] suggests that the corresponding undirected graphs may have a very different structure from the sparse graphs arising from the analysis of protein sequences. As mentioned in [15] the authors use several BHOSLIB benchmarks to compare LEAF with other methods; we have used the four largest datsets which have 1272 nodes (frb53-24), 1400 nodes (frb56-25), 1534 nodes (frb59-26) and 4000 nodes (frb100-40). With 1000 iterations, FastIndep outperforms LEAF on all of these benchmarks based on the results shown in Figure six of [15]. On the the largest dataset used running FastIndep 1000 times yields 850 maximal independent sets larger than the one found by LEAF, and on this data set outperforms three of the five methods discussed in [15]. However none of the methods discussed in [15] permit the generation of multiple maximal independent sets, which as we have argued earlier is a desirable feature when analyzing population stratification. These considerations suggest that FastIndep has some distinct advantages for creating multiple maximal independent subsets from panels of thousands of markers for cross-checking the analysis of population stratification.

As a final study with artificial data we check how closely the results of FastIndep match exact results on a set of graphs of size 150. We generate a number of random graphs with varying thresholds and check the size of the largest maximal independent set found by 1500 runs of FastIndep compared with the true sizes as obtained from the branch and bound methods of [12,13]. The results are shown in Table 4, the discrepancy between the size of the largest maximal independent set found by FastIndep and the true size appears to be largest for low connectivities and decreases with connectivity. In Table 4 (and in the rest of this paper) connectivity is defined to be the ratio of the number of edges actually present in the graph to the total number of edges that would be present if all nodes were connected. Thus connectivity is zero for a graph with no edges and 1 for a graph in which all possible edges are present. Sparse graphs have low connectivities, and for the random graphs generated by thresholding discussed earlier, the sum of the threshold value and the connectivity is approximately one. Thus the thresholds in Table 2 for which Primus outperforms FastIndep correspond to low connectivities, which is consistent with the results in Table 4 where the discrepancy between FastIndep and the branch and bound results is largest for small connectivities.

**Table 4:** Comparison with true maximum independentset size

**Connectivity**	**Largest set found**	**True size of maximum**
	**by FastIndep**	**independent set**
0.0976	33	37
0.203	22	23
0.304	15	17
0.398	12	14
0.503	10	10
0.596	9	9
0.700	7	7
0.798	6	6
0.899	4	4

Set sizes found by FastIndep for a graph of size 150 with varying connectivities.

### Results on real data

FastIndep has been used for finding unlinked markers in data sets with very complex Linkage Disequilibrium patterns such as [17] where the extent of Linkage Disequilbrium in the ancestral populations varies so much that there is no easy way to use map information alone to select a set of independent markers. Retaining all the markers in [17] for analyzing population stratification along the lines of STRUCTURE [18] leads to poor convergence, however the use of a maximal independent set of markers selected along the lines of [8] leads to much better results because the algorithm in [8] ignores map information in selecting markers. As an added feature, the randomized nature of the algorithm permits the generation of multiple different sets of markers which can be successively used in the analysis of population stratification to ensure that the final results are independent of the choice of the set of markers; this procedure has been followed in [17].

**Figure 1:**
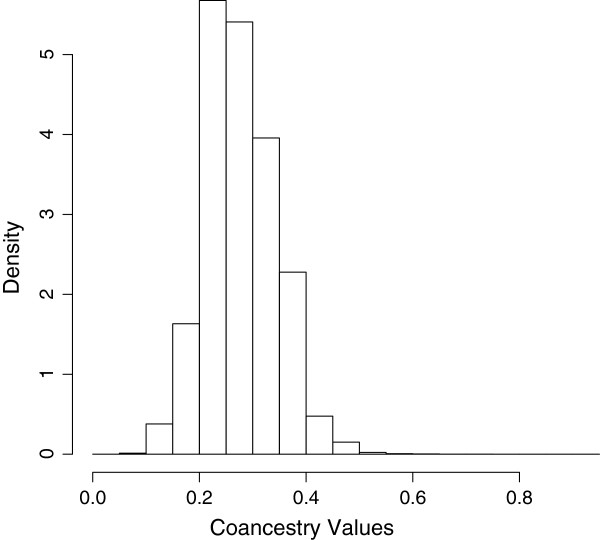
**Histogram of Coancestry Values.** Histogram of Coancestry Values of the Cattle Population.

FastIndep was applied on a genetic coancestry matrix estimated in a real cattle population. Molecular coancesty was obtained using the methods of [19] utilizing genotypes of 4057 animals with information available for 17 microsatellites that are normally used for parentage verification and parental assignment. These animals are the offspring of 2605 and 108 distinct dams and sires, respectively. Therefore, there are two dominant types of structures, full-sib and half-sib families are present in the data set. The distribution of coancestry values (Table 5 and Figure 1) ranged from 0.0147 to 0.7353 which is to be expected because as realized by [20] there are variations in the molecular relationship between pairs of individual having the same pedigree relationship. The other aspect that could be observed in this data was that the distribution of molecular coancestry values did not show a smooth pattern which is also in agreement with the presence of a structure in the population.

**Table 5:** Quantiles of data set 1

**Minimum**	**25%**	**Median**	**75%**	**Maximum**
0.0147	0.2353	0.2794	0.3235	0.7353

Quantile Distribution of coancestry values of the cattle data set.

The algorithm was run 1000 times using 7 thresholds corresponding to graphs with 4057 nodes and connectivities ranging from 7.29 × 10^-7^ to 0.999. On a laptop with a 2.3GHz processor, 4GB of RAM running MacOS and gcc version 4.2.1 the total running time was 533.836 seconds, 307.413 seconds were needed for one graph with connectivity 0.00142 and 1600 singletons, animals unrelated to all others. The distribution of maximal independent set sizes is shown in Table 6 for those thresholds for which there was substantial variation in the size of the maximal independent sets found.

**Table 6:** Maximal independent set size distribution

**Connectivity**	**Minimum**	**25%**	**Median**	**75%**	**Maximum**
0.899	3	5	5	6	8
0.346	40	48	50	52	76
0.0328	611	654	664	672	846
0.00142	2697	2740	2751	2762	2907
0.0000679	3854	3870	3874	3878	3895

Quantile Distribution of Maximal Independent Set Sizes of the cattle data set.

As an additional test on real data we have selected 5000 markers at random from the snps.10 dataset of the Bioconductor [21] snpStats package [22]. These markers have been selected after weakly filtering all markers present for a threshold in Minor Allele frequency (>0.05) and Hardy Weinberg Disequilibrium $\left(z_{\text{HWE}}^{2}<200\right)$. The linkage disequilibrium matrix between these markers was obtained using the methods in snpStats and FastIndep was used to find independent markers. A threshold value of *r*^2^ of 0.01 was used and the distribution in sizes of maximal independent sets is shown in Table 7. The choice of threshold was motivated by the fact that *r*^2^ is given by $\frac{\chi^{2}}{\left(2n\right)}$, where the number of individuals *n* is 1000, leading to a significance threshold of 7.7 × 10^-6^. The test statistic is choosen to be higher than those usually considered for *χ*^2^ tests on account of the large number of tests performed. The connectivity of the graph with this threshold is 0.27, which is much larger than the largest value of 0.03 reported in [15]. A less stringent significance threshold would lead to an even lower threshold value for *r*^2^ and a larger connectivity. Thus some of the applications for which FastIndep is designed give rise to graphs which have a very different structure from those discussed in [15]. However as mentioned earlier, identifying unrelated individuals among the subjects in a genetic association analysis study may require analyzing very much sparser graphs.

**Table 7:** Maximal independent set size distribution

**Minimum**	**25%**	**Median**	**75%**	**Maximum**
184	198	202	206	234

Quantile Distribution of Maximal Independent Set Sizes from LD matrix.

It is interesting to note that the connectivity of the graph arising from the LD matrix is in the regime where the discrepancy between the size of the largest maximal independent set found by FastIndep and exact answer may be small judging by the results in Table 4. This is consistent with the results in [17] where the LD matrix of a small subset of markers was used to check the discrepancy between the largest independent set found by FastIndep and the exact answer; the discrepancy was found to be small.

## Conclusions

We have introduced FastIndep, a stochastic heuristic for finding maximal independent sets on large undirected graphs. The results presented in this paper indicate that FastIndep provides an efficient and competitive heuristic for generating maximal independent sets even for graphs with thousands of nodes, sizes which can easily arise in modern genetic analyses. This is a valuable feature when selecting unrelated individuals or unrelated markers for analyzing population stratification. Indeed, modern biobanks may contain many tens of thousands of samples, so efficiently identifying unrelated samples in such datasets is crucial. Furthermore the capability of FastIndep to find multiple solutions with the same cardinality may shed light on the existence of multiple maximal independent sets which deterministic heuristics may fail to uncover. We have also argued that this capability of finding multiple maximal independent subsets provides a means for cross checking results obtained in the analysis of population stratification. Since the algorithm in FastIndep is heuristic, there is no guarentee that the set of largest cardinality found is the maximum independent set, or that the algorithm has discovered all distinct maximum independent sets. If there is some doubt, the program can be allowed to run more times in order to better explore the graph structure. Comparison with exact algorithms on smaller data sets suggest the discrepancy between the size of the largest maximal independent set found by FastIndep and size of the maximum independent set is largest when connectivities are small, *i.e.* for sparse graphs.

## Competing interests

The authors declare that they have no competing interests.

## Authors’ contributions

KJA programmed the algorithm, CD made many valuable suggestions which lead to a substantial improvements over the inital version of the program. Both authors have read and approved the final manuscript.
